# Safeguarding lymphatic identity: cooperative Erg and Fli1 activity in lymphatic vascular homeostasis

**DOI:** 10.1172/JCI203656

**Published:** 2026-03-02

**Authors:** Kelly de Korodi, Tatiana V. Petrova

**Affiliations:** 1 Department of Fundamental Oncology, University of Lausanne, Lausanne, Switzerland.; 2 Ludwig Institute for Cancer Research, Lausanne, Switzerland.; 3 ISREC – Swiss Institute for Experimental Cancer Research, School of Life Sciences, Ecole Polytechnique Fédérale de Lausanne, Lausanne, Switzerland.

## Abstract

Although transcriptional programs driving lymphatic endothelial cell (LEC) specification are being increasingly characterized, far less is known about the postnatal mechanisms that preserve lymphatic vessel identity and function. In this issue of the *JCI*, Yang et al. show that the E26 transformation-specific (ETS) transcription factors ETS-related gene (Erg) and Friend leukemia integration 1 (Fli1) cooperatively maintain adult LEC homeostasis by sustaining transcriptionally distinct LEC populations, vascular integrity, immune-vascular interactions, and repression of proinflammatory and prothrombotic gene programs. These findings extend the known roles of Erg and Fli1 beyond the blood endothelium and provide mechanistic insight into human lymphatic disease associated with Erg haploinsufficiency.

## Introduction

Cell identity is an actively maintained state. Across tissues, differentiated cells can lose or alter their fate following injury, disease, or disruption of key transcriptional regulators. In the vascular system, endothelial cells (ECs) adapt their phenotype to local mechanical, metabolic, and inflammatory cues (1, 2). Understanding not only how endothelial identities are established during development, but also how they are preserved in adult tissues is therefore essential for deciphering vascular disease mechanisms and for designing regenerative therapies.

The blood and lymphatic vascular systems are intimately interconnected and together ensure tissue fluid balance, immune surveillance, and dietary lipid transport (3). Lymphatic ECs (LECs) organize into a hierarchical system of capillary and collecting vessels that return interstitial fluid and immune cells to the venous circulation. These functional distinctions are reflected in transcriptional heterogeneity among LEC subsets, shaped by vessel type and tissue context (3–5).

Substantial progress has been made in defining the transcriptional networks that specify lymphatic identity during development. Prox1 functions as the principal regulator of LEC fate, required throughout life to suppress blood endothelial programs and maintain lymphatic identity (6–8). Recent work (9) has identified the E26 transformation-specific (ETS) transcription factor (TF) Etv2 as a critical upstream regulator of LEC specification, demonstrating that a transient population of Etv2^+^ mesenchymal angioblasts can directly give rise to LECs without transitioning through a venous endothelial state. Mechanistically, Etv2, together with other ETS TFs, directly binds *cis*-regulatory elements within the *Prox1* locus in these early angioblasts, implicating ETS factors in the earliest steps of lymphatic lineage commitment (9). Additional TFs, including Foxc2, Gata2, Nfatc1, and Foxp2, orchestrate the specialization of collecting vessels and lymphatic valve formation (10–14). Mutations in many of these regulators cause primary lymphedema (LD) in humans, underscoring the clinical relevance of lymphatic transcriptional control (15).

ETS-related gene (Erg) and Friend leukemia integration 1 (Fli1) TFs have well established roles in blood endothelium. Erg is required for vascular development, EC stability, and hemostasis, while Fli1 plays overlapping roles during embryogenesis (16–21). Although deletion of either factor alone in adult ECs is not lethal, it results in organ-specific vascular defects, whereas Erg and Fli1 together cooperate to maintain blood vascular EC (BEC) identity and suppress inflammatory gene programs (20). Whether this cooperative transcriptional control extends to lymphatic endothelium had remained unclear. Yang et al. (22) provide compelling evidence that Erg and Fli1 are critical for preserving adult LEC identity, underscoring their central roles in maintaining LEC fate, specialization, and function.

## Erg and Fli1 cooperate to preserve lymphatic integrity

Inducible deletion of *Erg* and *Fli1* in adult mouse LECs resulted in rapid lethality accompanied by a profound loss of lymphatic function, including impaired drainage and severe lymph stasis across multiple vascular beds. In contrast, deletion of *Erg* or *Fli1* alone did not produce overt lymphatic abnormalities or lethality within the 1-month postdeletion analysis period, underscoring their functional cooperation in mature lymphatic vessels (22).

**Figure 1:**
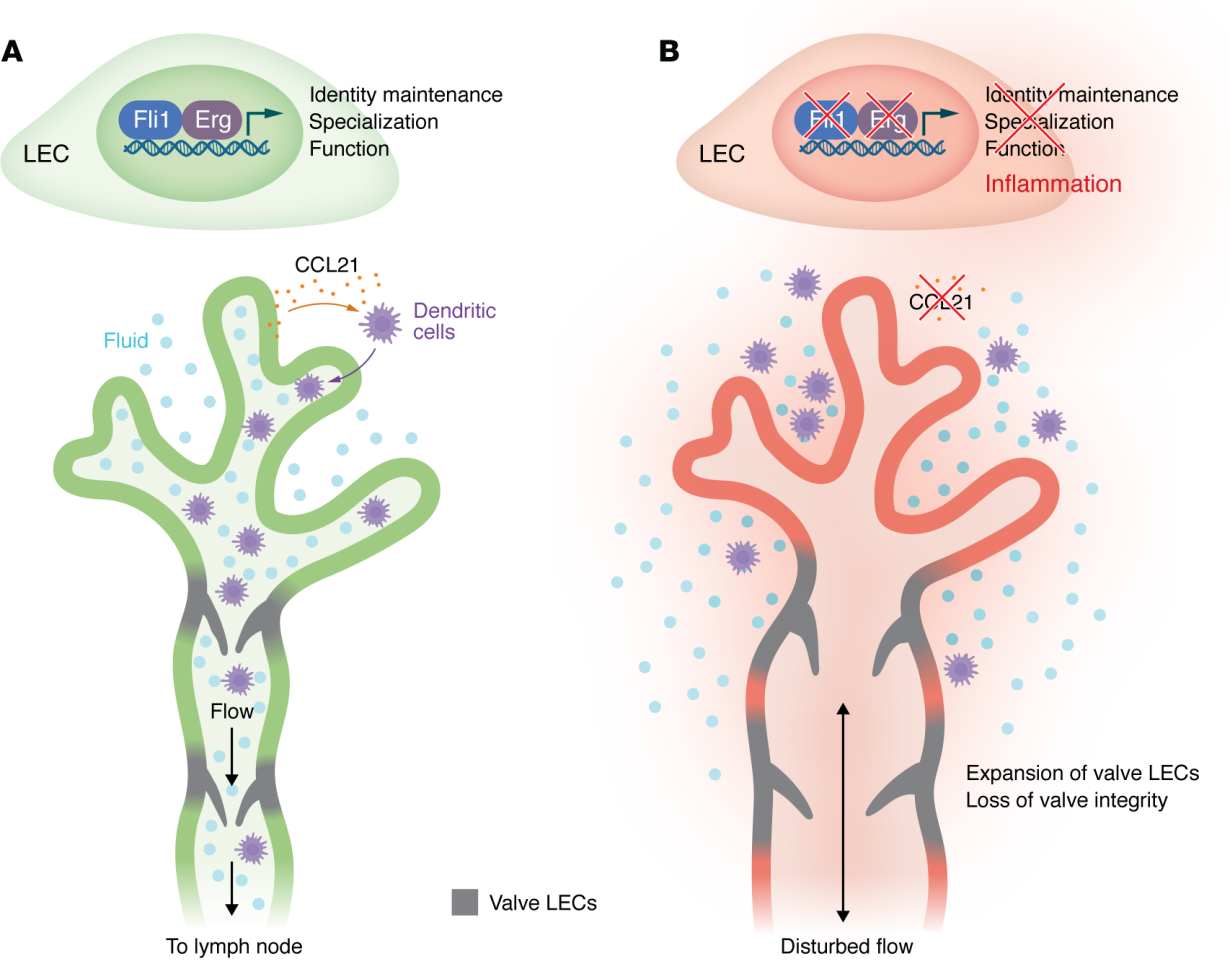
Cooperative action of Erg and Fli1 maintains lymphatic endothelial identity, specialization, and function. (**A**) Adult lymphatic vasculature consists of capillary lymphatic vessels, which take up interstitial fluid and serve as the entry point for DCs, and collecting vessels, which transport lymph to draining lymph nodes. Intraluminal valves ensure directional lymph flow. (**B**) In Yang et al. (22), loss of Erg and Fli1 resulted in the breakdown of the core lymphatic endothelial transcriptional program, including downregulation of *Ccl21*, encoding a key chemoattractant for DC migration. LECs also aberrantly acquired valve-like characteristics and activated proinflammatory and prothrombotic gene programs. Functionally, the combined loss of Erg and Fli1 disrupted essential lymphatic functions, leading to compromised lymph transport, impaired DC trafficking, and progressive degeneration of lymphatic valves.

At the transcriptional level, scRNA-seq revealed a collapse of LEC identity following combined Erg and Fli1 (Erg-Fli1) loss, with widespread downregulation of core lymphatic markers and disruption of normal LEC subset-specific gene expression programs. Among the most prominent changes was the rapid loss of *Ccl21* expression, a key mediator of immune cell trafficking through lymphatics (Figure 1). Mechanistically, the authors demonstrated direct binding of Erg to regulatory elements upstream of the *Ccl21* promoter. Erg-Fli1–deficient LECs also exhibited inappropriate activation of valve-associated transcriptional programs, with ectopic expression of genes normally restricted to valve LECs (Figure 1). This transcriptional mispatterning was accompanied by structural disruption of lymphatic valves. LECs from Erg-Fli1 double-knockout mice were also shifted toward a pathological activation state, characterized by induction of prothrombotic factors, including *Serpine1*, and inflammatory chemokines, such as *Ccl2*. Notably, lymph coagulation and inflammatory activation have been described in human disease settings, including fatal COVID-19, where excessive neutrophil accumulation along lymphatics and neutrophil extracellular trap formation contribute to lymphatic clotting (23).

Beyond adult homeostasis, Erg and Fli1 were required during embryonic lymphatic vascular development. Combined deletion during embryogenesis caused severe lymphatic defects and edema, with Erg alone playing a dominant role during developmental lymphangiogenesis. In adult lymphatic injury models, loss of Erg and Fli1 impaired lymphatic regeneration and exacerbated disease severity (22).

## Implications for human disease and unresolved paradoxes

These findings have direct relevance to human pathology. Recent genetic studies have identified loss-of-function Erg variants in individuals with primary LD, providing the first direct link between Erg and human lymphatic disease (24, 25). Patients harboring these variants present with peripheral LD, yet the mechanisms underlying lymphatic failure were previously unclear. The work by Yang et al. (22) now offers a compelling mechanistic framework, demonstrating that Erg is essential for maintaining lymphatic identity and vascular integrity in embryonic development and adulthood.

Importantly, the context of Erg function differs between primary and secondary LD. Human primary LD associated with Erg haploinsufficiency likely reflects an essential, largely Fli1-independent role for Erg during embryonic lymphatic development, consistent with the developmental defects observed upon Erg loss alone (22). In contrast, the cooperative requirement for Erg and Fli1 uncovered in adult lymphatic vessels may point to a distinct mechanism more relevant to secondary LD, in which inflammatory or injury-induced downregulation of both TFs could compromise lymphatic identity, repair, and function.

At the same time, Erg haploinsufficiency has also been linked to hematological malignancies (26) and more tentatively to cardiovascular diseases (20, 27), raising an intriguing question: Why do lymphatic phenotypes dominate in surviving patients despite the well-established importance of Erg in BECs? Several nonmutually exclusive explanations may account for this discrepancy. First, transcriptional redundancy within the ETS family is perhaps greater in BECs than in LECs, with many Erg target genes in BECs cobound by multiple ETS factors (28), potentially buffering the effects of Erg loss. Second, lymphatic vessels may operate closer to a critical threshold of Erg activity, such that modest reductions preferentially disrupt lymphatic function while sparing blood vascular homeostasis. Third, and perhaps most compelling, is survivorship bias: individuals with Erg haploinsufficiency who reach postnatal life may represent a select population in whom compensatory mechanisms preserve essential blood vascular functions, whereas more severe vascular defects may be embryonically lethal.

The elegant study by Yang et al. not only defines Erg and Fli1 as core transcriptional gatekeepers of LEC identity, but also creates new questions. The precise molecular interactions between Erg/Fli1 and established lymphatic regulators and how they shape lymphatic compartment-specific programs and chromatin accessibility require further investigation. Notably, the forced expression of Erg and Fli1 in mesenchymal stromal cells supports the formation of functional perfusable blood vascular networks in vivo (20), underscoring that Erg and Fli1 alone are insufficient to confer lymphatic identity. Lymphatic valve-specific gene expression is tightly regulated by biomechanical forces (12), and the aberrant overexpression of these markers in the absence of Erg and Fli1 suggests that the activity of these TFs may be required to restrain inappropriate mechanosensitive signaling in LECs. In the disease context, it is critical to determine how Erg and Fli1 are modulated during lymphatic injury, LD, and inflammation and whether targeted restoration of their activity could promote repair, maintain lymphatic identity, and improve disease outcomes.

## Funding support

- European Union’s Horizon 2020 research and innovation program Theralymph (grant 874708 to TVP).
- Swiss National Science Foundation (CR32I3_166326, 310030_212387, 310030_197878, and 237945 to TVP).
- Swiss Cancer League (KFS-5685-08-2022 and KLS-6555-08-2025 to TVP).
- ISREC Foundation.
- Novartis Foundation.
- Fond’action Foundation.

## Version 1 03/02/2026

Electronic publication
